# *Leishmania* Infection Induces MicroRNA hsa-miR-346 in Human Cell Line-Derived Macrophages

**DOI:** 10.3389/fmicb.2018.01019

**Published:** 2018-05-17

**Authors:** Aurora Diotallevi, Mauro De Santi, Gloria Buffi, Marcello Ceccarelli, Fabrizio Vitale, Luca Galluzzi, Mauro Magnani

**Affiliations:** ^1^Department of Biomolecular Sciences, Section of Biotechnology, University of Urbino, Fano, Italy; ^2^Department of Biomolecular Sciences, Section of Hygiene, University of Urbino, Urbino, Italy; ^3^Istituto Zooprofilattico Sperimentale della Sicilia, Palermo, Italy

**Keywords:** *Leishmania*, *Viannia*, macrophages, U937 cells, THP-1 cells, hsa-miR-346, RFX1, IL18

## Abstract

Leishmaniasis is an anthropo-zoonotic disease caused by various *Leishmania* species. The clinical manifestations of the disease vary according to the species and host characteristics. *Leishmania* infection leads to subversion/modulation of the host’s innate immune response and cellular metabolic pathways. In the last years, it has been shown that many host cell gene expression and signaling pathways are targeted by *Leishmania* to subvert host defenses (e.g., oxidative damage, immune activation, antigen presentation, apoptosis) and allow parasite survival and replication. However, the molecular mechanisms triggered by the parasite are not fully elucidated. The role of miRNA has recently been evaluated in human or murine macrophages infected with *Leishmania* (*Leishmania*) *major*, *L.* (*L.*) *donovani* or *L.* (*L.*) *amazonensis.* However, no literature exists regarding miRNA dysregulation in host cells infected with *L.* (*L.*) *infantum* or *L.* (*Viannia*) species. Since we previously showed that *L.* (*L.*) *infantum* infection induced unfolded protein response (UPR) in macrophages, we focused on miR-346, which has been shown to be induced by the UPR-activated transcription factor sXBP1 and has a potential role in the modulation of the immune response. Macrophages differentiated from U937 and/or THP-1 human monocytic cells were infected with four *L.* (*L.*) *infantum* strain/clinical isolates and one *L*. (*V.*) sp. clinical isolate. A significant upregulation of miR-346 (*p* < 0.05) was observed in infections with all the *Leishmania* species tested. Moreover, RFX1 (a miR-346 predicted target gene) was found to be significantly downregulated (*p* < 0.05) after 48h infection, and miR-346 was found to have a role in this downregulation. The induction of miR-346 in macrophages infected with *L*. (*L*.) *infantum* and *L*. (*V.*) sp., reported here for the first time, could play a role in regulating macrophage functions since several MHC- or interferon-associated genes are among the targets of this miRNA. Hence, miR-346 could be considered an attractive anti-*Leishmania* drug target.

## Introduction

Leishmaniasis is an anthropo-zoonotic disease caused by various *Leishmania* species belonging to the subgenera *Leishmania* and *Viannia*. Affecting about 12 million people, it is considered a global health problem due to its diffusion in Europe, Africa, Asia (Old World) as well as in the Americas (New World) (Alvar et al., 2012; Akhoundi et al., 2016). It has been estimated that there are 0.7–1 million new cases of the disease every year causing 20,000–30,000 deaths (World Health Organization, 2018). *Leishmania* infection can lead to various forms of the disease, which vary according to the *Leishmania* species and host characteristics. These forms include cutaneous leishmaniasis (CL), visceral leishmaniasis (VL) or MCL. *Leishmania* parasites are transmitted by female sandflies to their mammalian hosts. Once infection occurs, the parasites enter phagocytic cells (mainly macrophages). Here, the parasite subverts the host’s innate immune response and metabolic pathways, surviving and replicating in the phagolysosomal environment (Arango Duque and Descoteaux, 2015; Podinovskaia and Descoteaux, 2015). *Leishmania* strategies for evasion of host defenses include: (i) suppression of inflammation by upregulation of host peroxisome proliferator-activated receptor-gamma (PPARγ) and protein tyrosine phosphatases, (ii) prevention of macrophage activation by interference with the Janus kinase/signal transducers and activators of transcription (JAK/STAT) pathway, (iii) inhibition of reactive oxygen species (ROS) generation by preventing NADPH oxidase assembly (Podinovskaia and Descoteaux, 2015). Moreover, antigen cross-presentation through major histocompatibility complex (MHC) class I has been shown to be inhibited by metalloprotease GP63-mediated cleavage of VAMP8 (Vesicle Associated Membrane Protein 8) in murine bone marrow-derived macrophages infected with *Leishmania* (*Leishmania*) *major* or *L.* (*L.*) *donovani* promastigotes. Disruption of VAMP8 prevented NADPH oxidase assembly on phagosomes, altering their degradative properties and inhibiting *Leishmania* antigen presentation (Matheoud et al., 2013). Interestingly, the antigen presentation through MHC class II molecules is also compromised in *Leishmania* infection, as shown by reduced IFNγ-induced transcription of MHC class II molecules in bone marrow-derived murine macrophages infected with *L.* (*L.*) *donovani* amastigotes (Matte and Descoteaux, 2010).

Recently, it has been shown that host cell endoplasmic reticulum (ER) stress and unfolded protein response (UPR) is triggered by *L.* (*L.*) *amazonensis* and *L.* (*L.*) *infantum* parasites and that the activation of this pathway plays an important role in infection progression (Dias-Teixeira et al., 2016, 2017; Galluzzi et al., 2016). The UPR in mammalian cells is driven by three ER protein: PKR-like endoplasmic reticulum kinase (PERK), inositol-requiring enzyme 1 (IRE1) and activating transcription factor 6 (ATF6) (Galluzzi et al., 2017). PERK activation induces a global translation attenuation by phosphorylation of eukaryotic initiation factor 2α (eIF2α), and the expression of genes involved in antioxidant response via phosphorylation of the transcription factor NRF2. IRE1 induces the splicing of the X-box binding protein 1 (XBP1) mRNA (sXBP1), allowing the translation of an active transcription factor, which leads to the expression of genes that expand the folding capacity of the ER and promote ER-associated degradation (ERAD). ATF6 is a proteolytically activated transcription factor that induces the transcription of XBP1 and contributes to optimization of the UPR by controlling a number of genes related to ERAD and lipid synthesis.

MicroRNAs (miRNAs) are short non-coding RNAs that regulate the expression of target genes post-transcriptionally. MiRNAs recognize target mRNA exploiting a small sequence situated at positions 2–7 at the 5′ end of the miRNA, which is called the seed region. In animals, the miRNA–mRNA interaction is semi-complementary, therefore a single miRNA can interact with many transcripts and a single transcript can be targeted by many miRNAs. The interaction miRNA–mRNA leads to translation inhibition and mRNA destabilization (Cloonan, 2015). Notably, several miRNAs have been shown to be involved in UPR signaling (Maurel and Chevet, 2013). Moreover, miRNAs play an important role in the activation of macrophages and in the regulation of phagocytosis and apoptosis (Wei and Schober, 2016). There is growing evidence that Apicomplexa parasites promote modifications in the host miRNA population, highlighting the important role that these molecules play in parasite-host interactions. Indeed, alterations in host cell miRNAs have been described in human and/or murine macrophages infected with *L.* (*L.*) *major*, *L.* (*L.*) *donovani* or *L.* (*L.*) *amazonensis* (Lemaire et al., 2013; Geraci et al., 2015; Muxel et al., 2017; Tiwari et al., 2017). In particular, miR-146a, which is involved in modulation of both the innate and adaptive immune responses (Labbaye and Testa, 2012), was found to be dysregulated in *L*. (*L*.) *major* infection (Lemaire et al., 2013; Geraci et al., 2015). However, to the best of our knowledge, there is no data in the literature regarding miRNA dysregulation in human host cells infected with *L.* (*L.*) *infantum* or *L.* (*Viannia*) sp.

Interestingly, it has been shown that miR-346 is induced by sXBP1 during ER stress in various cell types (Bartoszewski et al., 2011). MiR-346 plays a role in the modulation of the immune response by targeting MHC-associated genes or interferon-inducible genes (Bartoszewski et al., 2011). Moreover, miR-346 has been shown to act as a pro-survival factor under ER stress in HeLa cells, by promoting autophagy. In particular, miR-346 was shown to promote mitophagy, thus reducing the ROS level in the cell (Guo et al., 2018). In light of these findings, and considering that *Leishmania* infection elicited sXBP1, we investigated miR-346 expression in human cell line-derived macrophages infected with *Leishmania* parasites.

## Materials and Methods

### Parasite Isolation and Cultivation

*Leishmania* parasites were cultivated at 26–28°C in Evans’ Modified Tobie’s Medium (EMTM) (Castelli et al., 2014). Every 5 days stationary promastigote were transferred to fresh medium (ratio 1:5). *L.* (*L.*) *infantum* MHOM/TN/80/IPT1 (WHO international reference strain) and human clinical isolate 31U were provided by the World Organisation for Animal Health (OIE) Reference Laboratory National Reference Center for Leishmaniasis (C.Re.Na.L.) located in Palermo (Italy). Two *L.* (*L.*) *infantum* clinical isolates (isolate 1, isolate 2) had been previously obtained from lymph node aspirates of two infected dogs from an *L.* (*L.*) *infantum* endemic region in Italy (Galluzzi et al., 2016). A further *Leishmania* sp. clinical isolate was obtained from a pharyngo-laryngeal biopsy during routine diagnosis of a human patient with suspect MCL. This isolate was recognized as *L.* (*V.*) sp. by previously described qPCR assays followed by HRM analysis from 82 to 90°C (Ceccarelli et al., 2014, 2017) and PCR product sequencing. For parasite isolation, the biopsied materials were placed in 5 ml of sterile liquid Tobie medium and centrifuged at 350 *× g* 10 min at 4°C. Supernatants were discarded and tissue pellets were disrupted by pipetting in a 1 ml sterile Tobie medium. A 0.5 ml volume was added to a flask containing solid Tobie’s medium. After 5–7 days at 26°C, the presence of parasites was established by phase-contrast microscopic observation at 40X magnification.

### Cell Culture-Derived Macrophages and Infection

The human monocytic cell lines U937 (ATCC CRL-1593.2) and THP-1 (ECACC 88081201) were routinely cultured in RPMI-1640 medium supplemented with 10% heat-inactivated Fetal Bovine Serum (FBS), 2 mM L-glutamine, 1% penicillin/streptomycin at 37°C and 5% CO_2_. To differentiate monocytes into macrophage-like cells, 6 × 10^5^ cells were stimulated with 10 ng/ml phorbol myristic acid (PMA) for 72 h in 35 mm dishes. All cell culture reagents were purchased from Sigma Aldrich. The *Leishmania* reference strain and clinical isolates stationary promastigotes were used to infect U937 and THP-1-derived macrophages with a parasite-to-cell ratio of 10:1 as described (Galluzzi et al., 2016). To synchronize the entrance of parasites into cells, dishes were centrifuged at 450 × *g* for 3 min. This step favored contact between parasites and macrophages, making infection more homogeneous. Infections were repeated twice (one dish per infection). After 24 or 48 h, cells were washed to remove free parasites and directly lysed for downstream analyses. The infection index was calculated in cells stained with Hoechst dye by multiplying the percentage of infected macrophages by the average number of parasites per macrophage. At least 300 total macrophages were counted for each infection time. As an ER stress positive control, cells were treated with 0.5 μg/ml tunicamycin for 4 h or 1mM dithiothreitol (DTT) for 1 h.

### THP-1 Transfection With miR-346 Inhibitor

To test the impact of hsa-miR-346 on expression of predicted target genes following *Leishmania* spp. infection, THP-1 derived macrophages were transfected with the miR-346 inhibitor and the miRNA inhibitor negative control (Integrated DNA Technologies) at 50 nM final concentration. THP-1 derived macrophages were transfected using the HiPerFect Transfection Reagent (Qiagen) according to the manufacturer’s protocol. After 6 h of incubation with the transfection complexes, cells were infected following the protocol described above.

### RNA Isolation and cDNA Synthesis

Macrophage-like cells were directly lysed with 700 μl of QIAzol^®^ Lysis Reagent (Qiagen, Hilden, Germany). Total RNA extraction was performed with the miRNeasy Mini Kit (Qiagen, Hilden, Germany) following the manufacturer’s instructions. To quantify extracted RNA a NanoVue PlusTM spectrophotometer (GE Healthcare Life Sciences, Piscataway, NJ, United States) was used and the integrity/quality was assessed by 1% agarose gel stained with GelRed (Biotium, Hayward, CA, United States). For gene expression, 500 ng total RNA were reverse transcribed using PrimeScriptTM RT Master Mix (Perfect Real Time) (Takara Bio Inc.) according to the manufacturer’s instructions. The cDNA synthesis for three microRNAs (miR-126, miR-146a, and miR-346) was performed by TaqMan^TM^ MicroRNA Reverse Transcription Kit (Applied Biosystems) using 12.5 ng of total RNA.

### Quantitative Real-Time PCR

The expression of sXBP1, RFX1, TAP1, IL18, and BCAP31 was monitored by qPCR using RT^2^ SYBR Green ROX FAST Mastermix (Qiagen) with primers listed in **Table 1**, in a RotorGene 6000 instrument (Corbett life science, Sydney, Australia). The amount corresponding to 2 ng of total RNA used for cDNA synthesis was loaded into each PCR tube. For all targets, the amplification conditions were as follows: 95°C for 10 min, 40 cycles at 95°C for 15 s and 60°C for 50 s. A non-template control was included in duplicate for each primer pair reaction as negative control. To exclude the presence of non-specific products or primer dimers, a melting curve analysis from 65 to 95°C was performed at the end of each run. B2M (beta-2-microglobulin) and/or GUSB (Beta-D-Glucuronidase) were used as reference genes. In order to evaluate the expression of three microRNAs (miR-126, miR-146a, and miR-346), the qPCR analyses were performed on a 7500 Real Time PCR System (Applied Biosystems) using specific Taqman small RNA assays (Applied Biosystems). The reactions were performed in a final volume of 20 μl with the following thermal protocol: 95°C for 10 min, 40 cycles at 95°C for 15 s and 60°C for 1 min. The data were normalized against small nucleolar RNAs (RNU44 and/or RNU48). The relative expression levels were calculated using the 2^-ΔΔC_t_^ method (Pfaffl, 2001).

**Table 1:** Primers used for gene expression analysis.

Target mRNA	Accession number	Forward primer (5′-3′)	Reverse primer (5′-3′)	Reference
sXBP1	NM_001079539	CTGAGTCCGCAGCAGGT	TGTCCAGAATGCCCAACAGG	Galluzzi et al., 2016
RFX1	NM_002918	CTCCCTGAACCCCCTGGA	CCAGCCGCCAGTGAGATG	
TAP1	NM_000593	CCCAGAAGCCAACTATGGAGG	AGCCTCGTCTACCTCTGTGT	
IL18	NM_001562	TGACTGTAGAGATAATGCACCCC	AGTTACAGCCATACCTCTAGGC	
BCAP31	NM_001139457	TGCTGTCCTTCCTGCTTAGA	CACTAGCACTCTCCGCCTG	
B2M	NM_004048	ACTGAATTCACCCCCACTGA	CCTCCATGATGCTGCTTACA	Zhang et al., 2005
GUSB	NM_000181	GCTACTACTTGAAGATGGTGATCG	AGTTAGAGTTGCTCACAAAGGTC	Galluzzi et al., 2016

### *In Silico* Analysis of miRNA Targets

The miRWalk 2.0 (Dweep and Gretz, 2015) and miRTarBase (Chou et al., 2018) databases for experimentally validated microRNA-target interactions were used for miR-346 target identification. Gene ontology analysis was performed using GOrilla (Eden et al., 2009). The miR-346 target genes were inputted as the “target set,” and human genes with NCBI gene ID downloaded from the Ensembl database were inputted as “background set.”

### Statistical Analysis

Data were analyzed by unpaired *t*-test using Prism software version 5.0 (GraphPad, San Diego, CA, United States). All data are presented as the mean ± standard deviation (SD). The unpaired *t*-test was used because the data consisted of the means of two independent groups. A two-tailed *p*-value was used to determine if the two groups were significantly different.

## Results

### ER Stress Induces Upregulation of miR-346 in Human Monocytic Cell Line

**FIGURE 1:**
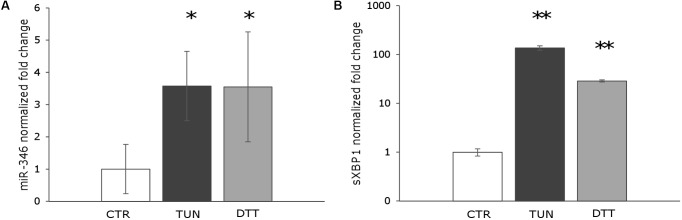
miR-346 is upregulated following ER stress in human monocytic cell line. **(A)** miR-346 expression in THP-1 cells treated with 0.5 μg/ml tunicamycin for 4 h (dark gray) or 1 mM dithiothreitol for 1 h (light gray) (*n* = 4). **(B)** sXBP1 expression in the same cells used to monitor miR-346 expression (*n* = 4). CTR, untreated cells; TUN, cells treated with tunicamycin; DTT, cells treated with dithiothreitol. ^∗^*p* < 0.05; ^∗∗^*p* < 0.01.

It has been shown that miR-346 is induced in response to ER stress in different cell types (Calu-3, HeLa, primary glioblastoma, and primary astrocytoma cells) and that its expression is induced by sXBP1 (Bartoszewski et al., 2011). To explore the potential role of miR-346 in *Leishmania* infection, we first determined whether miR-346 could be induced following ER stress in a monocytic cell line. THP-1 cells were treated with tunicamycin and DTT as ER stressors and sXBP1 and miR-346 expression were monitored as described in methods. Tunicamycin is an inhibitor of the UDP-N-acetylglucosamine:dolichyl-phosphate N-acetylglucosamine phosphotransferase (GPT). Treatment with tunicamycin inhibits glycosylation of newly synthesized proteins, triggering ER stress. DTT is a strong reducing agent that inhibits disulfide-bond formation, rapidly leading to ER stress (Oslowski and Urano, 2011). The expression of miR-346 and sXBP1 significantly increased in treated cells in response to all tested ER stressor molecules (**Figure 1**). These results were consistent with data reported in the literature using other cell types and established a rational basis for the assessment of miR-346 expression in our infection model.

### miR-346 is Upregulated in U937 and THP-1-Derived Macrophages Infected With *L. (L.) infantum* and *L. (V.)* sp. strains

**FIGURE 2:**
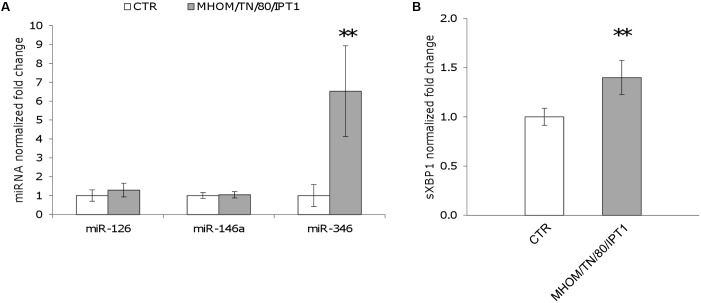
miR-346 is upregulated in U937-derived macrophages infected with *L.* (*L.*) *infantum.* **(A)** miR-126, miR-146a, and miR-346 expression in cells infected for 24 h with *L.* (*L.*) *infantum* MHOM/TN/80/IPT1 strain (gray bars) relative to control cells (white bars). **(B)** sXBP1 expression in infected cells (gray bars) compared to control cells (white bars). Data are represented as the mean ± SD of two experiments (*n* = 4). CTR: non-infected cells. ^∗∗^*p* < 0.01.

**FIGURE 3:**
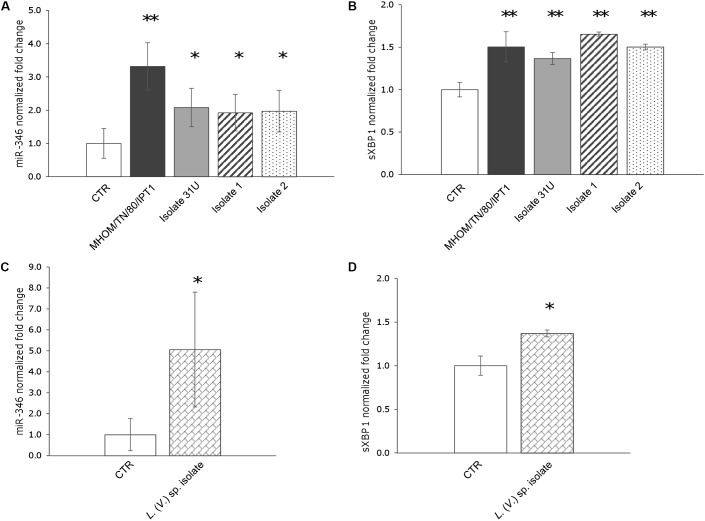
miR-346 is upregulated in THP-1-derived macrophages infected with *L.* (*L.*) *infantum* and *L.* (*V.*) sp. **(A)** miR-346 expression in cells infected for 24 h with *L.* (*L.*) *infantum* MHOM/TN/80/IPT1 strain (dark gray bars) or clinical isolate 31U (light gray bars), isolate 1 (dashed bars), isolate 2 (dotted bars). **(B)** sXBP1 expression in the same cells used to monitor miR-346 expression. **(C)** miR-346 expression in cells infected for 24 h with *L.* (*V.*) sp. isolate (bricked bars). **(D)** sXBP1 expression in infected cells (bricked bars) compared to control cells (white bars). Data are represented as the mean ± SD of two experiments (*n* = 4). CTR: non-infected cells. ^∗^*p* < 0.05; ^∗∗^*p* < 0.01.

First, the expression of miR-346, as well as miR-146a and miR-126, was monitored in U937-derived macrophages infected with *L.* (*L.*) *infantum* MHOM/TN/80/IPT1 as described in methods. MiR-146a has been shown to be dysregulated in *L.* (*L.*) *major* infection (Lemaire et al., 2013; Geraci et al., 2015), while miR-126 (mainly involved in angiogenesis and in the modulation of dendritic cell function) (Ferretti and La Cava, 2014) was not expected to have a role in macrophages infected with *Leishmania*. Twenty-four hours after infection, miR-146a did not appear significantly dysregulated, confirming previous findings in human primary macrophages infected with *L. major* for 24 h (Lemaire et al., 2013). Moreover, the expression of miR-126 was not affected either. However, a significant upregulation of miR-346 was detected after 24 h infection with *L.* (*L.*) *infantum* (**Figure 2A**). Furthermore, a mild but significant upregulation of sXBP1 was also observed in infected cells, which is consistent with previous results (Galluzzi et al., 2016) (**Figure 2B**). The expression of miR-346 was then further investigated in THP-1-derived macrophages infected with *L.* (*L.*) *infantum* MHOM/TN/80/IPT1, as well as human clinical isolate 31U and canine clinical isolates 1 and 2. All strains induced miR-346 and sXBP1 expression (**Figures 3A,B**). Interestingly, similar results were obtained in cells infected with a *Leishmania* clinical isolate belonging to the subgenus *Viannia* (**Figures 3C,D**). It is also noteworthy that miR-346 was poorly expressed (*C*_t_ > 32) in monocytic cell lines compared to miR-146a and miR-126. The infection indexes with *L.* (*L.*) *infantum* MHOM/TN/80/IPT1, isolate 31U and *L.* (*V.*) sp. isolate are reported in Supplementary Figure S1.

### *In Silico* Identification of miR-346 Targets

In order to identify the transcripts targeted by miR-346 in infected macrophages, we used the validated module of miRWalk 2.0 and miRTarBase. The joined results allowed us to identify 76 predicted target genes (Supplementary Table S1) that are significantly associated with 33 gene ontology (biological process) terms (Supplementary Table S2). Many of these terms are related to immune system processes, pointing to a potential role of this miRNA in curbing immune response in *Leishmania* infection.

### RFX1 and IL18 Are Downregulated 48 h After Infection With *L. (L.) infantum* and *L. (V.)* sp.

**FIGURE 4:**
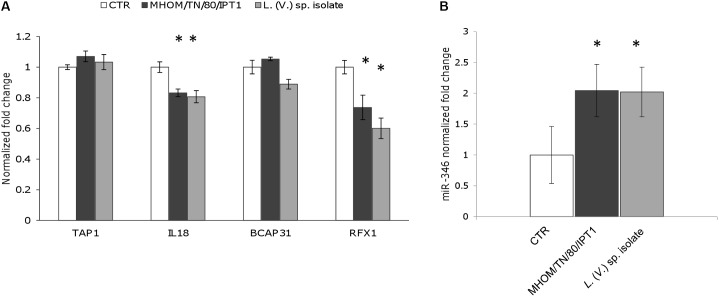
RFX1 and IL18 are downregulated after 48 h infection with *L.* (*L.*) *infantum* and *L.* (*V*.) sp. **(A)** The expression of four miR-346 predicted target genes (TAP1, BCAP31, RFX1, IL18), associated with MHC and interferon gamma production, was evaluated in THP-1-derived macrophages after 48h infection with *L.* (*L.*) *infantum* MHOM/TN/80/IPT1 strain (dark gray bars) and *L.* (*V.*) sp. isolate (light gray bars) (*n* = 4). **(B)** miR-346 expression in THP-1-derived macrophages after 48 h infection with *L.* (*L.*) *infantum* MHOM/TN/80/IPT1 strain (dark gray bars) and *L.* (*V.*) sp. isolate (light gray bars) (*n* = 4). CTR: non-infected cells. ^∗^*p* < 0.05.

To monitor the possible effects of miR-346 upregulation, we selected four genes (TAP1, BCAP31, RFX1, IL18) associated with MHC and interferon gamma production from the 76 identified genes, and their expression was monitored by qPCR in cells infected with *Leishmania* parasites. THP-1 and/or U937-derived macrophages infected with *L.* (*L.*) *infantum* MHOM/TN/80/IPT1, clinical isolate 31U, canine clinical isolates 1 and 2, and *L.* (*V.*) sp. isolate did not show any significant downregulation of the four predicted target genes after 24 h (not shown). The same results were obtained with THP-1-derived macrophages treated with the ER stressors tunicamycin and DTT (not shown). On the other hand, after 48h infection, IL18 and RFX1 appeared significantly downregulated in THP-1-derived macrophages infected with both *L.* (*L.*) *infantum* MHOM/TN/80/IPT1 and *L.* (*V.*) sp. isolate (**Figure 4A**). Importantly, miR-346 (**Figure 4B**), as well as sXBP1 (not shown), still appeared significantly upregulated after 48 h infection. It is noteworthy that the basal expression of RFX1 in THP-1-derived macrophages was lower than that of other genes (Supplementary Figure S2), making this gene more prone to be regulated by the low expressed miR-346. TAP1 has been shown to be regulated by miR-346 in other cellular models, including HeLa cells (Bartoszewski et al., 2011), but it was not found downregulated in our infection model. To account for the lack of TAP1 regulation in our model, the basal expression of TAP1 in HeLa cells and in THP-1-derived macrophages was investigated. We found that TAP1 was about three times more expressed in THP-1-derived macrophages, making this transcript less susceptible to downregulation by miR-346 (Supplementary Figure S3).

### RFX1 mRNA Is a Target of miR-346 in Infected THP-1-Derived Macrophages

**FIGURE 5:**
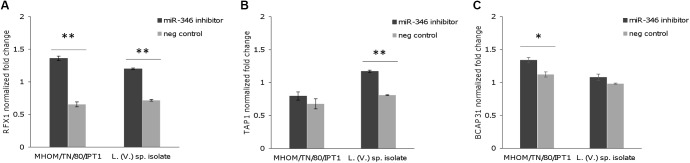
Expression of predicted miR-346 target genes in infected THP-1-derived macrophages transfected with miR-346 inhibitor. **(A–C)** Relative mRNA levels of RFX1 **(A)**, TAP1 **(B)**, and BCAP31 **(C)** were evaluated in THP-1-derived macrophages after 48h infection with *L.* (*L.*) *infantum* MHOM/TN/80/IPT1 strain and *L.* (*V.*) sp. isolate. Before infection, cells were transfected with the miR-346 inhibitor (dark gray bars) or with the miRNA inhibitor negative control (light gray bars) (*n* = 4). Expression levels were normalized against transfected non-infected cells. ^∗^*p* < 0.05; ^∗∗^*p* < 0.01.

To further investigate whether miR-346 is involved in post-transcriptional regulation of its predicted target genes in *Leishmania* infection, we transfected THP-1 derived macrophages with a miR-346 inhibitor. After 48 h infection with *L*. (*L*.) *infantum* MHOM/TN/80/IPT1 strain and *L*. (*V*.) sp. isolate, relative mRNA levels of IL18, RFX1, TAP1, BCAP31 were evaluated by qPCR. The transfection with the miR-346 inhibitor resulted in a significant increase in relative amount of RFX1 mRNA, compared to cells transfected with miRNA inhibitor negative control (**Figure 5A**). This increase was also partly evident for TAP1 and BCAP31 mRNA (**Figures 5B,C**) while it was not detected in IL18 mRNA (not shown).

## Discussion

Many intracellular parasites have been shown to modify host cell miRNA expression profiles. For instance, miR-146a and/or miR-155 were found to have a role in host cell response to *Toxoplasma gondii* infection (Cannella et al., 2014), cerebral malaria (Barker et al., 2017) and *L.* (*L.*) *major* infection (Lemaire et al., 2013). Moreover, *L.* (*L.*) *donovani* was shown to target Dicer1 and to downregulate miR-122 in mouse liver, leading to increased parasite burden (Ghosh et al., 2013). More recently, using next generation sequencing-, microarray - or qPCR-based approaches, several works demonstrated that *L. (L.) major, L. (L.) donovani* or *L. (L.) amazonensis* infection changes the miRNA expression profile in macrophages (Frank et al., 2015; Geraci et al., 2015; Muxel et al., 2017; Tiwari et al., 2017) and dendritic cells (Geraci et al., 2015). However, it is difficult to identify a common miRNA signature elicited by *Leishmania* infection due to the heterogeneity of the experimental models/conditions (i.e., differences in methods for small RNAs extraction, detection/quantification; differences regarding infection time points, *Leishmania* species/strains and host cells). For example, miR-146a, known to have an anti-inflammatory role (Wei and Schober, 2016), was found to be upregulated in early (3 h) infection in human primary macrophages (Lemaire et al., 2013) and appeared downregulated after 24 h infection in human dendritic cells (Geraci et al., 2015).

A number of studies have demonstrated changes in miRNA levels following ER stress, with several miRNAs induced or repressed by UPR effectors (Malhi, 2014). In particular, the expression of miR-346, involved in the modulation of the immune response, was shown to be mediated by sXBP1 (Bartoszewski et al., 2011). Since XBP1 splicing and modulation of immune response are among the effects induced by *Leishmania* infection, we considered miR-346 an interesting target to investigate in our infection model. A significant upregulation of miR-346 was detected, along with the induction of sXBP1, in both U937 and THP-1-derived macrophages infected with four *L*. (*L*.) *infantum* strains and/or one *L.* (*V*.) sp. isolate. This finding could help us to link the previously observed induction of UPR (Galluzzi et al., 2016) with the parasite elusion mechanisms of the cell-mediated immune response. Indeed, several miR-346 targets are associated with immune functions (e.g., MHC- or interferon-associated genes) (Supplementary Table S2).

A significant dysregulation of miR-346 has never been reported in *Leishmania* infection. This may be due to the characteristics of the infection models or to the low expression level of this miRNA. In the latter case, the limited dynamic range of microarray-based approaches may have hampered the detection/quantification of this miRNA. On the other hand, approaches based on qPCR can allow detection and quantification of low-level targets, thanks to the high sensitivity and dynamic range of this technique. However, the qPCR-based assays used in previous works did not include primers specific for this particular miRNA (Lemaire et al., 2013).

The magnitude of the miR-346 induction in response to ER stress inducers (tunicamycin or DTT) and *Leishmania* infection was comparable, although sXBP1 mRNA was enormously induced in tunicamycin or DTT treated cells compared to infected cells. In other words, the magnitude of the induction of sXBP1 did not appear to be proportionally related to the induction of miR-346. This might depend on the time point analyzed or on the post-transcriptional regulation of sXBP1. Alternatively, it could reflect a more complex regulation of miR-346 transcription, in which some other factors may be involved.

To assess the potential effects of infection-induced miR-346, four genes (TAP1, IL18, BCAP31, RFX1) were selected among the predicted targets of miR-346 based on information in the literature and on their function in relation to the immune response. The expression of selected genes was monitored by qPCR after 24 and 48 h of infection. TAP1 is one of the best characterized miR-346 target and it is involved in the pumping of degraded cytosolic peptides across the endoplasmic reticulum into the membrane-bound compartment where MHC class I molecules assemble. Reduced TAP1 mRNA and protein levels have been associated with decreased MHC class I antigen presentation (Lankat-Buttgereit and Tampé, 2002; Bartoszewski et al., 2011). IL18 is a proinflammatory cytokine, involved in the Th1 immune response, which synergizes with IL12 and stimulates interferon gamma production in Th1 cells (Arango Duque and Descoteaux, 2014). BCAP31 encodes for a transmembrane protein of the endoplasmic reticulum that is involved in the transport of membrane proteins from the endoplasmic reticulum to the Golgi, in caspase 8- mediated apoptosis and in the export of MHC class I molecules from the ER (Abe et al., 2009). RFX1 encodes for a transcription factor that regulates a variety of genes involved in immunity and cancer, including the MHC class II genes. Only RFX1 and IL18 genes showed significant downregulation after 48 h infection. The fact that BCAP31 and TAP1 expression were not affected might be explained by the low amount of miR-346 in cell line-derived macrophages. Indeed, based on *C*_t_ evaluation in non-infected cells, we observed that BCAP31 was the most represented transcript, followed by TAP1, IL18, and RFX1. The basal expression of IL18 and RFX1 was about 20 and 200 times lower than that of BCAP31 (Supplementary Figure S2). Therefore, IL18 and RFX1 were more likely to be regulated by the poorly represented miR-346 in our infection model. It is noteworthy that upregulation of sXBP1 and miR-346, and downregulation of IL18 and RFX1 were observed in infected cells regardless of *Leishmania* subgenus, pointing to a possible common pathogenic mechanism in the *L.* (*Leishmania*) and *L.* (*Viannia*) parasites.

It is well known that *Leishmania* interferes with the production of cytokines, such as IL12, and with antigen presentation by dendritic cells and macrophages to counteract the host immune response (Liu and Uzonna, 2012; Cecílio et al., 2014). Hence, the downregulation of IL18 and RFX1 could be part of the molecular mechanisms induced by the parasite to survive and proliferate in host cells. In fact, IL18 has a role in synergizing with IL12 for IFN-γ production and establishment of Th1 immunity, while RFX1 may play a role in the regulation of MHC class II expression. We hypothesized that this downregulation could be mediated by miR-346. However, experiments with the miR-346 inhibitor indicated that miR-346 has a role in the regulation of RFX1 mRNA, but not in IL18 mRNA. Moreover, an increase in TAP1 and BCAP31 mRNA was also partly observed in infected cells treated with miR-346 inhibitor, suggesting a role of miR-346 in counteracting the upregulation of these genes during infection.

If the role of miR-346 in the modulation of the immune response is confirmed, this molecule could be an attractive anti-*Leishmania* drug target. Indeed, its silencing could lead to a more efficient immune response and/or to early death of infected macrophages, since miR-346 was also shown to protect cells from death under ER stress.

In summary, miR-346 was found to be upregulated in two human cell line-derived macrophages infected with four different strains/isolates of *L. (L.) infantum*, as well as one *L. (V*.) sp. isolate, pointing to a possible common pathogenic mechanism in the *Leishmania* and *Viannia* subgenera. Since miR-346 has been shown to have a role in both the modulation of the immune response and in cell survival under ER stress, antisense strategy against this target could be considered for anti-*Leishmania* approaches.

## Author Contributions

AD and LG contributed to design of the work, acquisition, and analysis/interpretation of data, drafting the work and revising it critically. MDS, GB, and MC contributed to acquisition, analysis/interpretation of data, and revising the manuscript critically. FV and MM contributed to analysis/interpretation of data, and revising the manuscript critically. All authors approved the final manuscript.

## Conflict of Interest Statement

The authors declare that the research was conducted in the absence of any commercial or financial relationships that could be construed as a potential conflict of interest.

## Abbreviations

DTT: dithiothreitol
HRM: high resolution melt
MCL: mucocutaneous leishmaniasis
