# Supplementary material for: Leishmania Infection Induces MicroRNA hsa-miR-346 in Human Cell Line-Derived Macrophages
Source: Front Microbiol. 2018 May 17;9:1019. doi: 10.3389/fmicb.2018.01019 (PMC5966562; doi:10.3389/fmicb.2018.01019)
Supplement: Supplementary file 2 [file Table_2.DOCX]

**Table S2. Biological process gene ontology (GO) terms significantly enriched among the 76 validated mir-346 target genes.**

| **GO term** | **Description** | **P-value^1^** | **FDR q-value^2^** | **Enrichment (N, B, n, b)^3^** | **Genes** |
| --- | --- | --- | --- | --- | --- |
| GO:0071364 | cellular response to epidermal growth factor stimulus | 4.88E-4 | 3.92E-1 | 19.46 (18238,38,74,3) | PDPK1 - 3-phosphoinositide dependent protein kinase-1  ZFP36 - zfp36 ring finger protein  CFLAR - casp8 and fadd-like apoptosis regulator |
| GO:0070849 | response to epidermal growth factor | 6.57E-4 | 3.85E-1 | 17.60 (18238,42,74,3) | PDPK1 - 3-phosphoinositide dependent protein kinase-1  ZFP36 - zfp36 ring finger protein  CFLAR - casp8 and fadd-like apoptosis regulator |
| GO:0018022 | peptidyl-lysine methylation | 2.44E-4 | 4.13E-1 | 13.14 (18238,75,74,4) | KMT2D - lysine (k)-specific methyltransferase 2d  PRDM16 - pr domain containing 16  EZH1 - enhancer of zeste homolog 1 (drosophila)  SETD4 - set domain containing 4 |
| GO:0016571 | histone methylation | 4.31E-4 | 4.38E-1 | 11.33 (18238,87,74,4) | KMT2D - lysine (k)-specific methyltransferase 2d  PRDM16 - pr domain containing 16  EZH1 - enhancer of zeste homolog 1 (drosophila)  PRMT5 - protein arginine methyltransferase 5 |
| GO:0002474 | antigen processing and presentation of peptide antigen via MHC class I | 5.11E-4 | 3.9E-1 | 10.83 (18238,91,74,4) | PSMD1 - proteasome (prosome, macropain) 26s subunit, non-atpase, 1  PSMA3 - proteasome (prosome, macropain) subunit, alpha type, 3  BCAP31 - b-cell receptor-associated protein 31  TAP1 - transporter 1, atp-binding cassette, sub-family b (mdr/tap) |
| GO:0006479 | protein methylation | 1.6E-4 | 6.09E-1 | 9.78 (18238,126,74,5) | KMT2D - lysine (k)-specific methyltransferase 2d  PRDM16 - pr domain containing 16  EZH1 - enhancer of zeste homolog 1 (drosophila)  PRMT5 - protein arginine methyltransferase 5  SETD4 - set domain containing 4 |
| GO:0008213 | protein alkylation | 1.6E-4 | 4.88E-1 | 9.78 (18238,126,74,5) | KMT2D - lysine (k)-specific methyltransferase 2d  PRDM16 - pr domain containing 16  EZH1 - enhancer of zeste homolog 1 (drosophila)  PRMT5 - protein arginine methyltransferase 5  SETD4 - set domain containing 4 |
| GO:0038093 | Fc receptor signaling pathway | 9.6E-5 | 1E0 | 8.13 (18238,182,74,6) | PDPK1 - 3-phosphoinositide dependent protein kinase-1  PSMD1 - proteasome (prosome, macropain) 26s subunit, non-atpase, 1  PSMA3 - proteasome (prosome, macropain) subunit, alpha type, 3  BTK - bruton agammaglobulinemia tyrosine kinase  LIMK1 - lim domain kinase 1  CD247 - cd247 molecule |
| GO:0006367 | transcription initiation from RNA polymerase II promoter | 4.43E-4 | 4.22E-1 | 7.85 (18238,157,74,5) | NR6A1 - nuclear receptor subfamily 6, group a, member 1  MED16 - mediator complex subunit 16  RXRB - retinoid x receptor, beta  TEAD2 - tea domain family member 2  NR2F6 - nuclear receptor subfamily 2, group f, member 6 |
| GO:0050851 | antigen receptor-mediated signaling pathway | 5.87E-4 | 4.26E-1 | 7.38 (18238,167,74,5) | PDPK1 - 3-phosphoinositide dependent protein kinase-1  PSMD1 - proteasome (prosome, macropain) 26s subunit, non-atpase, 1  PSMA3 - proteasome (prosome, macropain) subunit, alpha type, 3  BTK - bruton agammaglobulinemia tyrosine kinase  CD247 - cd247 molecule |
| GO:0002699 | positive regulation of immune effector process | 6.36E-4 | 4.04E-1 | 7.25 (18238,170,74,5) | PDPK1 - 3-phosphoinositide dependent protein kinase-1  IL18 - interleukin 18 (interferon-gamma-inducing factor)  GPI - glucose-6-phosphate isomerase  BTK - bruton agammaglobulinemia tyrosine kinase  PVR - poliovirus receptor |
| GO:0051348 | negative regulation of transferase activity | 1.18E-4 | 9E-1 | 5.37 (18238,367,74,8) | PDPK1 - 3-phosphoinositide dependent protein kinase-1  PSMD1 - proteasome (prosome, macropain) 26s subunit, non-atpase, 1  ZFP36 - zfp36 ring finger protein  PSMA3 - proteasome (prosome, macropain) subunit, alpha type, 3  GSK3B - glycogen synthase kinase 3 beta  LIMK1 - lim domain kinase 1  TERF1 - telomeric repeat binding factor (nima-interacting) 1  MAPK8IP1 - mitogen-activated protein kinase 8 interacting protein 1 |
| GO:0002429 | immune response-activating cell surface receptor signaling pathway | 8.91E-4 | 4.24E-1 | 5.36 (18238,276,74,6) | PDPK1 - 3-phosphoinositide dependent protein kinase-1  PSMD1 - proteasome (prosome, macropain) 26s subunit, non-atpase, 1  PSMA3 - proteasome (prosome, macropain) subunit, alpha type, 3  BTK - bruton agammaglobulinemia tyrosine kinase  LIMK1 - lim domain kinase 1  CD247 - cd247 molecule |
| GO:0031349 | positive regulation of defense response | 2.47E-4 | 3.43E-1 | 4.82 (18238,409,74,8) | PSMD1 - proteasome (prosome, macropain) 26s subunit, non-atpase, 1  PDPK1 - 3-phosphoinositide dependent protein kinase-1  IL18 - interleukin 18 (interferon-gamma-inducing factor)  CNPY3 - canopy fgf signaling regulator 3  PSMA3 - proteasome (prosome, macropain) subunit, alpha type, 3  BTK - bruton agammaglobulinemia tyrosine kinase  ETS1 - v-ets avian erythroblastosis virus e26 oncogene homolog 1  PVR - poliovirus receptor |
| GO:0002757 | immune response-activating signal transduction | 7.37E-4 | 4.16E-1 | 4.68 (18238,369,74,7) | PDPK1 - 3-phosphoinositide dependent protein kinase-1  PSMD1 - proteasome (prosome, macropain) 26s subunit, non-atpase, 1  CNPY3 - canopy fgf signaling regulator 3  PSMA3 - proteasome (prosome, macropain) subunit, alpha type, 3  BTK - bruton agammaglobulinemia tyrosine kinase  LIMK1 - lim domain kinase 1  CD247 - cd247 molecule |
| GO:1903706 | regulation of hemopoiesis | 4.28E-4 | 4.66E-1 | 4.44 (18238,444,74,8) | PSMD1 - proteasome (prosome, macropain) 26s subunit, non-atpase, 1  KMT2D - lysine (k)-specific methyltransferase 2d  IL18 - interleukin 18 (interferon-gamma-inducing factor)  ZFP36 - zfp36 ring finger protein  PSMA3 - proteasome (prosome, macropain) subunit, alpha type, 3  BTK - bruton agammaglobulinemia tyrosine kinase  ETS1 - v-ets avian erythroblastosis virus e26 oncogene homolog 1  LIF - leukemia inhibitory factor |
| GO:0050778 | positive regulation of immune response | 8.14E-4 | 4.14E-1 | 3.62 (18238,613,74,9) | PSMD1 - proteasome (prosome, macropain) 26s subunit, non-atpase, 1  PDPK1 - 3-phosphoinositide dependent protein kinase-1  IL18 - interleukin 18 (interferon-gamma-inducing factor)  CNPY3 - canopy fgf signaling regulator 3  PSMA3 - proteasome (prosome, macropain) subunit, alpha type, 3  BTK - bruton agammaglobulinemia tyrosine kinase  LIMK1 - lim domain kinase 1  PVR - poliovirus receptor  CD247 - cd247 molecule |
| GO:0002684 | positive regulation of immune system process | 2.39E-4 | 4.56E-1 | 3.30 (18238,895,74,12) | PSMD1 - proteasome (prosome, macropain) 26s subunit, non-atpase, 1  PDPK1 - 3-phosphoinositide dependent protein kinase-1  IL18 - interleukin 18 (interferon-gamma-inducing factor)  CNPY3 - canopy fgf signaling regulator 3  PSMA3 - proteasome (prosome, macropain) subunit, alpha type, 3  BTK - bruton agammaglobulinemia tyrosine kinase  GPI - glucose-6-phosphate isomerase  ETS1 - v-ets avian erythroblastosis virus e26 oncogene homolog 1  LIMK1 - lim domain kinase 1  LIF - leukemia inhibitory factor  PVR - poliovirus receptor  CD247 - cd247 molecule |
| GO:0018193 | peptidyl-amino acid modification | 6.49E-4 | 3.96E-1 | 3.16 (18238,858,74,11) | PDPK1 - 3-phosphoinositide dependent protein kinase-1  KMT2D - lysine (k)-specific methyltransferase 2d  USP22 - ubiquitin specific peptidase 22  PRDM16 - pr domain containing 16  EZH1 - enhancer of zeste homolog 1 (drosophila)  BTK - bruton agammaglobulinemia tyrosine kinase  PRMT5 - protein arginine methyltransferase 5  GSK3B - glycogen synthase kinase 3 beta  ASGR2 - asialoglycoprotein receptor 2  LIF - leukemia inhibitory factor  SETD4 - set domain containing 4 |
| GO:0002682 | regulation of immune system process | 4.58E-4 | 3.88E-1 | 2.62 (18238,1409,74,15) | IL18 - interleukin 18 (interferon-gamma-inducing factor)  ZFP36 - zfp36 ring finger protein  PSMA3 - proteasome (prosome, macropain) subunit, alpha type, 3  CNPY3 - canopy fgf signaling regulator 3  LIMK1 - lim domain kinase 1  LIF - leukemia inhibitory factor  KMT2D - lysine (k)-specific methyltransferase 2d  PDPK1 - 3-phosphoinositide dependent protein kinase-1  PSMD1 - proteasome (prosome, macropain) 26s subunit, non-atpase, 1  GPI - glucose-6-phosphate isomerase  BTK - bruton agammaglobulinemia tyrosine kinase  IFNGR2 - interferon gamma receptor 2 (interferon gamma transducer 1)  ETS1 - v-ets avian erythroblastosis virus e26 oncogene homolog 1  PVR - poliovirus receptor  CD247 - cd247 molecule |
| GO:0045595 | regulation of cell differentiation | 2.47E-4 | 3.77E-1 | 2.55 (18238,1642,74,17) | IL18 - interleukin 18 (interferon-gamma-inducing factor)  PRDM16 - pr domain containing 16  ZFP36 - zfp36 ring finger protein  PSMA3 - proteasome (prosome, macropain) subunit, alpha type, 3  CFLAR - casp8 and fadd-like apoptosis regulator  LIMK1 - lim domain kinase 1  LIF - leukemia inhibitory factor  SSH3 - slingshot protein phosphatase 3  PSMD1 - proteasome (prosome, macropain) 26s subunit, non-atpase, 1  KMT2D - lysine (k)-specific methyltransferase 2d  GJC2 - gap junction protein, gamma 2, 47kda  INPP5J - inositol polyphosphate-5-phosphatase j  BTK - bruton agammaglobulinemia tyrosine kinase  PRMT5 - protein arginine methyltransferase 5  GSK3B - glycogen synthase kinase 3 beta  ETS1 - v-ets avian erythroblastosis virus e26 oncogene homolog 1  TEAD2 - tea domain family member 2 |
| GO:0048584 | positive regulation of response to stimulus | 1.32E-4 | 6.72E-1 | 2.41 (18238,2044,74,20) | IL18 - interleukin 18 (interferon-gamma-inducing factor)  PSMA3 - proteasome (prosome, macropain) subunit, alpha type, 3  CNPY3 - canopy fgf signaling regulator 3  PPP2R1B - protein phosphatase 2, regulatory subunit a, beta  CFLAR - casp8 and fadd-like apoptosis regulator  LIMK1 - lim domain kinase 1  LIF - leukemia inhibitory factor  MAPK8IP1 - mitogen-activated protein kinase 8 interacting protein 1  PIK3R3 - phosphoinositide-3-kinase, regulatory subunit 3 (gamma)  PDPK1 - 3-phosphoinositide dependent protein kinase-1  PSMD1 - proteasome (prosome, macropain) 26s subunit, non-atpase, 1  KMT2D - lysine (k)-specific methyltransferase 2d  HRK - harakiri, bcl2 interacting protein (contains only bh3 domain)  BCAP31 - b-cell receptor-associated protein 31  BTK - bruton agammaglobulinemia tyrosine kinase  PRMT5 - protein arginine methyltransferase 5  GSK3B - glycogen synthase kinase 3 beta  ETS1 - v-ets avian erythroblastosis virus e26 oncogene homolog 1  PVR - poliovirus receptor  CD247 - cd247 molecule |
| GO:0006357 | regulation of transcription by RNA polymerase II | 6.17E-4 | 4.09E-1 | 2.28 (18238,1945,74,18) | IL18 - interleukin 18 (interferon-gamma-inducing factor)  PRDM16 - pr domain containing 16  ZFP36 - zfp36 ring finger protein  PSMA3 - proteasome (prosome, macropain) subunit, alpha type, 3  MED16 - mediator complex subunit 16  POU2F1 - pou class 2 homeobox 1  RXRB - retinoid x receptor, beta  LIF - leukemia inhibitory factor  FOXF2 - forkhead box f2  RFX1 - regulatory factor x, 1 (influences hla class ii expression)  PSMD1 - proteasome (prosome, macropain) 26s subunit, non-atpase, 1  NR6A1 - nuclear receptor subfamily 6, group a, member 1  KMT2D - lysine (k)-specific methyltransferase 2d  BCOR - bcl6 corepressor  EZH1 - enhancer of zeste homolog 1 (drosophila)  ETS1 - v-ets avian erythroblastosis virus e26 oncogene homolog 1  TEAD2 - tea domain family member 2  NR2F6 - nuclear receptor subfamily 2, group f, member 6 |
| GO:0006793 | phosphorus metabolic process | 7.53E-4 | 4.1E-1 | 2.24 (18238,1978,74,18) | IL18 - interleukin 18 (interferon-gamma-inducing factor)  ZFP36 - zfp36 ring finger protein  PSMA3 - proteasome (prosome, macropain) subunit, alpha type, 3  LIMK1 - lim domain kinase 1  GALT - galactose-1-phosphate uridylyltransferase  LIF - leukemia inhibitory factor  MAPK8IP1 - mitogen-activated protein kinase 8 interacting protein 1  PIK3R3 - phosphoinositide-3-kinase, regulatory subunit 3 (gamma)  PDPK1 - 3-phosphoinositide dependent protein kinase-1  SSH3 - slingshot protein phosphatase 3  PSMD1 - proteasome (prosome, macropain) 26s subunit, non-atpase, 1  INPP5J - inositol polyphosphate-5-phosphatase j  SBF1 - set binding factor 1  TSTA3 - tissue specific transplantation antigen p35b  BTK - bruton agammaglobulinemia tyrosine kinase  CKB - creatine kinase, brain  GPI - glucose-6-phosphate isomerase  GSK3B - glycogen synthase kinase 3 beta |
| GO:0009892 | negative regulation of metabolic process | 7.73E-4 | 4.06E-1 | 1.96 (18238,2887,74,23) | PRDM16 - pr domain containing 16  ZFP36 - zfp36 ring finger protein  PSMA3 - proteasome (prosome, macropain) subunit, alpha type, 3  CFLAR - casp8 and fadd-like apoptosis regulator  POU2F1 - pou class 2 homeobox 1  APLP2 - amyloid beta (a4) precursor-like protein 2  LIMK1 - lim domain kinase 1  LIF - leukemia inhibitory factor  FOXF2 - forkhead box f2  TERF1 - telomeric repeat binding factor (nima-interacting) 1  MAPK8IP1 - mitogen-activated protein kinase 8 interacting protein 1  PDPK1 - 3-phosphoinositide dependent protein kinase-1  PSMD1 - proteasome (prosome, macropain) 26s subunit, non-atpase, 1  NR6A1 - nuclear receptor subfamily 6, group a, member 1  KMT2D - lysine (k)-specific methyltransferase 2d  BCOR - bcl6 corepressor  BSCL2 - berardinelli-seip congenital lipodystrophy 2 (seipin)  INPP5J - inositol polyphosphate-5-phosphatase j  GPI - glucose-6-phosphate isomerase  GSK3B - glycogen synthase kinase 3 beta  PRMT5 - protein arginine methyltransferase 5  RPL30 - ribosomal protein l30  NR2F6 - nuclear receptor subfamily 2, group f, member 6 |
| GO:0048856 | anatomical structure development | 4.44E-4 | 3.99E-1 | 1.95 (18238,3154,74,25) | LIMK1 - lim domain kinase 1  LIF - leukemia inhibitory factor  CCHCR1 - coiled-coil alpha-helical rod protein 1  IL11RA - interleukin 11 receptor, alpha  PDPK1 - 3-phosphoinositide dependent protein kinase-1  KMT2D - lysine (k)-specific methyltransferase 2d  BCOR - bcl6 corepressor  USP22 - ubiquitin specific peptidase 22  GJC2 - gap junction protein, gamma 2, 47kda  GPI - glucose-6-phosphate isomerase  BTK - bruton agammaglobulinemia tyrosine kinase  ETS1 - v-ets avian erythroblastosis virus e26 oncogene homolog 1  CD164 - cd164 molecule, sialomucin  PRDM16 - pr domain containing 16  CFLAR - casp8 and fadd-like apoptosis regulator  FOXF2 - forkhead box f2  SLC39A1 - solute carrier family 39 (zinc transporter), member 1  EZH1 - enhancer of zeste homolog 1 (drosophila)  CKB - creatine kinase, brain  GSK3B - glycogen synthase kinase 3 beta  PRMT5 - protein arginine methyltransferase 5  ASGR2 - asialoglycoprotein receptor 2  CRELD1 - cysteine-rich with egf-like domains 1  TEAD2 - tea domain family member 2  NR2F6 - nuclear receptor subfamily 2, group f, member 6 |
| GO:0044271 | cellular nitrogen compound biosynthetic process | 6E-4 | 4.16E-1 | 1.92 (18238,3216,74,25) | TERF1 - telomeric repeat binding factor (nima-interacting) 1  RFX1 - regulatory factor x, 1 (influences hla class ii expression)  NR6A1 - nuclear receptor subfamily 6, group a, member 1  PDPK1 - 3-phosphoinositide dependent protein kinase-1  BDH2 - 3-hydroxybutyrate dehydrogenase, type 2  KMT2D - lysine (k)-specific methyltransferase 2d  BCOR - bcl6 corepressor  USP22 - ubiquitin specific peptidase 22  BTK - bruton agammaglobulinemia tyrosine kinase  ETS1 - v-ets avian erythroblastosis virus e26 oncogene homolog 1  PRDM16 - pr domain containing 16  MED16 - mediator complex subunit 16  POU2F1 - pou class 2 homeobox 1  RXRB - retinoid x receptor, beta  FOXF2 - forkhead box f2  ZNF417 - zinc finger protein 417  EZH1 - enhancer of zeste homolog 1 (drosophila)  TSTA3 - tissue specific transplantation antigen p35b  POLD3 - polymerase (dna-directed), delta 3, accessory subunit  RAD51AP1 - rad51 associated protein 1  PRMT5 - protein arginine methyltransferase 5  RPL30 - ribosomal protein l30  TEAD2 - tea domain family member 2  GGT5 - gamma-glutamyltransferase 5  NR2F6 - nuclear receptor subfamily 2, group f, member 6 |
| GO:1901576 | organic substance biosynthetic process | 2.3E-4 | 5E-1 | 1.82 (18238,4197,74,31) | IL18 - interleukin 18 (interferon-gamma-inducing factor)  TERF1 - telomeric repeat binding factor (nima-interacting) 1  RFX1 - regulatory factor x, 1 (influences hla class ii expression)  PIK3R3 - phosphoinositide-3-kinase, regulatory subunit 3 (gamma)  KMT2D - lysine (k)-specific methyltransferase 2d  NR6A1 - nuclear receptor subfamily 6, group a, member 1  PDPK1 - 3-phosphoinositide dependent protein kinase-1  BDH2 - 3-hydroxybutyrate dehydrogenase, type 2  BCOR - bcl6 corepressor  USP22 - ubiquitin specific peptidase 22  INPP5J - inositol polyphosphate-5-phosphatase j  PTGS1 - prostaglandin-endoperoxide synthase 1 (prostaglandin g/h synthase and cyclooxygenase)  GPI - glucose-6-phosphate isomerase  BTK - bruton agammaglobulinemia tyrosine kinase  ETS1 - v-ets avian erythroblastosis virus e26 oncogene homolog 1  PRDM16 - pr domain containing 16  MED16 - mediator complex subunit 16  POU2F1 - pou class 2 homeobox 1  RXRB - retinoid x receptor, beta  FOXF2 - forkhead box f2  ZNF417 - zinc finger protein 417  EZH1 - enhancer of zeste homolog 1 (drosophila)  SBF1 - set binding factor 1  TSTA3 - tissue specific transplantation antigen p35b  POLD3 - polymerase (dna-directed), delta 3, accessory subunit  RAD51AP1 - rad51 associated protein 1  PRMT5 - protein arginine methyltransferase 5  TEAD2 - tea domain family member 2  RPL30 - ribosomal protein l30  GGT5 - gamma-glutamyltransferase 5  NR2F6 - nuclear receptor subfamily 2, group f, member 6 |
| GO:0044249 | cellular biosynthetic process | 3.46E-4 | 4.06E-1 | 1.81 (18238,4085,74,30) | TERF1 - telomeric repeat binding factor (nima-interacting) 1  RFX1 - regulatory factor x, 1 (influences hla class ii expression)  PIK3R3 - phosphoinositide-3-kinase, regulatory subunit 3 (gamma)  KMT2D - lysine (k)-specific methyltransferase 2d  NR6A1 - nuclear receptor subfamily 6, group a, member 1  PDPK1 - 3-phosphoinositide dependent protein kinase-1  BDH2 - 3-hydroxybutyrate dehydrogenase, type 2  BCOR - bcl6 corepressor  USP22 - ubiquitin specific peptidase 22  INPP5J - inositol polyphosphate-5-phosphatase j  PTGS1 - prostaglandin-endoperoxide synthase 1 (prostaglandin g/h synthase and cyclooxygenase)  GPI - glucose-6-phosphate isomerase  BTK - bruton agammaglobulinemia tyrosine kinase  ETS1 - v-ets avian erythroblastosis virus e26 oncogene homolog 1  PRDM16 - pr domain containing 16  MED16 - mediator complex subunit 16  POU2F1 - pou class 2 homeobox 1  RXRB - retinoid x receptor, beta  FOXF2 - forkhead box f2  ZNF417 - zinc finger protein 417  EZH1 - enhancer of zeste homolog 1 (drosophila)  SBF1 - set binding factor 1  TSTA3 - tissue specific transplantation antigen p35b  POLD3 - polymerase (dna-directed), delta 3, accessory subunit  RAD51AP1 - rad51 associated protein 1  PRMT5 - protein arginine methyltransferase 5  TEAD2 - tea domain family member 2  RPL30 - ribosomal protein l30  GGT5 - gamma-glutamyltransferase 5  NR2F6 - nuclear receptor subfamily 2, group f, member 6 |
| GO:0009058 | biosynthetic process | 3.07E-4 | 3.9E-1 | 1.79 (18238,4262,74,31) | IL18 - interleukin 18 (interferon-gamma-inducing factor)  TERF1 - telomeric repeat binding factor (nima-interacting) 1  RFX1 - regulatory factor x, 1 (influences hla class ii expression)  PIK3R3 - phosphoinositide-3-kinase, regulatory subunit 3 (gamma)  KMT2D - lysine (k)-specific methyltransferase 2d  NR6A1 - nuclear receptor subfamily 6, group a, member 1  PDPK1 - 3-phosphoinositide dependent protein kinase-1  BDH2 - 3-hydroxybutyrate dehydrogenase, type 2  BCOR - bcl6 corepressor  USP22 - ubiquitin specific peptidase 22  INPP5J - inositol polyphosphate-5-phosphatase j  PTGS1 - prostaglandin-endoperoxide synthase 1 (prostaglandin g/h synthase and cyclooxygenase)  GPI - glucose-6-phosphate isomerase  BTK - bruton agammaglobulinemia tyrosine kinase  ETS1 - v-ets avian erythroblastosis virus e26 oncogene homolog 1  PRDM16 - pr domain containing 16  MED16 - mediator complex subunit 16  POU2F1 - pou class 2 homeobox 1  RXRB - retinoid x receptor, beta  FOXF2 - forkhead box f2  ZNF417 - zinc finger protein 417  EZH1 - enhancer of zeste homolog 1 (drosophila)  SBF1 - set binding factor 1  TSTA3 - tissue specific transplantation antigen p35b  POLD3 - polymerase (dna-directed), delta 3, accessory subunit  RAD51AP1 - rad51 associated protein 1  PRMT5 - protein arginine methyltransferase 5  TEAD2 - tea domain family member 2  RPL30 - ribosomal protein l30  GGT5 - gamma-glutamyltransferase 5  NR2F6 - nuclear receptor subfamily 2, group f, member 6 |
| GO:0044238 | primary metabolic process | 2.11E-4 | 5.37E-1 | 1.45 (18238,8495,74,50) | IL18 - interleukin 18 (interferon-gamma-inducing factor)  APLP2 - amyloid beta (a4) precursor-like protein 2  LIMK1 - lim domain kinase 1  LIF - leukemia inhibitory factor  RFX1 - regulatory factor x, 1 (influences hla class ii expression)  HNRNPA3 - heterogeneous nuclear ribonucleoprotein a3  NR6A1 - nuclear receptor subfamily 6, group a, member 1  PIK3R3 - phosphoinositide-3-kinase, regulatory subunit 3 (gamma)  BDH2 - 3-hydroxybutyrate dehydrogenase, type 2  BCOR - bcl6 corepressor  INPP5J - inositol polyphosphate-5-phosphatase j  BTK - bruton agammaglobulinemia tyrosine kinase  GPI - glucose-6-phosphate isomerase  RAD54B - rad54 homolog b (s. cerevisiae)  PSMA3 - proteasome (prosome, macropain) subunit, alpha type, 3  MED16 - mediator complex subunit 16  CFLAR - casp8 and fadd-like apoptosis regulator  FOXF2 - forkhead box f2  ZNF417 - zinc finger protein 417  PSMD1 - proteasome (prosome, macropain) 26s subunit, non-atpase, 1  BSCL2 - berardinelli-seip congenital lipodystrophy 2 (seipin)  DCAF11 - ddb1 and cul4 associated factor 11  EZH1 - enhancer of zeste homolog 1 (drosophila)  TSTA3 - tissue specific transplantation antigen p35b  RAD51AP1 - rad51 associated protein 1  RPL30 - ribosomal protein l30  GGT5 - gamma-glutamyltransferase 5  GALT - galactose-1-phosphate uridylyltransferase  TERF1 - telomeric repeat binding factor (nima-interacting) 1  SETD4 - set domain containing 4  PDPK1 - 3-phosphoinositide dependent protein kinase-1  KMT2D - lysine (k)-specific methyltransferase 2d  SSH3 - slingshot protein phosphatase 3  USP22 - ubiquitin specific peptidase 22  PTGS1 - prostaglandin-endoperoxide synthase 1 (prostaglandin g/h synthase and cyclooxygenase)  ETS1 - v-ets avian erythroblastosis virus e26 oncogene homolog 1  ZFP36 - zfp36 ring finger protein  PRDM16 - pr domain containing 16  RXRB - retinoid x receptor, beta  POU2F1 - pou class 2 homeobox 1  MAPK8IP1 - mitogen-activated protein kinase 8 interacting protein 1  SBF1 - set binding factor 1  FNTB - farnesyltransferase, caax box, beta  POLD3 - polymerase (dna-directed), delta 3, accessory subunit  CKB - creatine kinase, brain  GSK3B - glycogen synthase kinase 3 beta  ASGR2 - asialoglycoprotein receptor 2  PRMT5 - protein arginine methyltransferase 5  TEAD2 - tea domain family member 2  NR2F6 - nuclear receptor subfamily 2, group f, member 6 |
| GO:0071704 | organic substance metabolic process | 8.22E-4 | 4.04E-1 | 1.39 (18238,8895,74,50) | IL18 - interleukin 18 (interferon-gamma-inducing factor)  APLP2 - amyloid beta (a4) precursor-like protein 2  LIMK1 - lim domain kinase 1  LIF - leukemia inhibitory factor  RFX1 - regulatory factor x, 1 (influences hla class ii expression)  HNRNPA3 - heterogeneous nuclear ribonucleoprotein a3  NR6A1 - nuclear receptor subfamily 6, group a, member 1  PIK3R3 - phosphoinositide-3-kinase, regulatory subunit 3 (gamma)  BDH2 - 3-hydroxybutyrate dehydrogenase, type 2  BCOR - bcl6 corepressor  INPP5J - inositol polyphosphate-5-phosphatase j  BTK - bruton agammaglobulinemia tyrosine kinase  GPI - glucose-6-phosphate isomerase  RAD54B - rad54 homolog b (s. cerevisiae)  PSMA3 - proteasome (prosome, macropain) subunit, alpha type, 3  MED16 - mediator complex subunit 16  CFLAR - casp8 and fadd-like apoptosis regulator  FOXF2 - forkhead box f2  ZNF417 - zinc finger protein 417  PSMD1 - proteasome (prosome, macropain) 26s subunit, non-atpase, 1  BSCL2 - berardinelli-seip congenital lipodystrophy 2 (seipin)  DCAF11 - ddb1 and cul4 associated factor 11  EZH1 - enhancer of zeste homolog 1 (drosophila)  TSTA3 - tissue specific transplantation antigen p35b  RAD51AP1 - rad51 associated protein 1  RPL30 - ribosomal protein l30  GGT5 - gamma-glutamyltransferase 5  GALT - galactose-1-phosphate uridylyltransferase  TERF1 - telomeric repeat binding factor (nima-interacting) 1  SETD4 - set domain containing 4  PDPK1 - 3-phosphoinositide dependent protein kinase-1  KMT2D - lysine (k)-specific methyltransferase 2d  SSH3 - slingshot protein phosphatase 3  USP22 - ubiquitin specific peptidase 22  PTGS1 - prostaglandin-endoperoxide synthase 1 (prostaglandin g/h synthase and cyclooxygenase)  ETS1 - v-ets avian erythroblastosis virus e26 oncogene homolog 1  ZFP36 - zfp36 ring finger protein  PRDM16 - pr domain containing 16  RXRB - retinoid x receptor, beta  POU2F1 - pou class 2 homeobox 1  MAPK8IP1 - mitogen-activated protein kinase 8 interacting protein 1  SBF1 - set binding factor 1  FNTB - farnesyltransferase, caax box, beta  POLD3 - polymerase (dna-directed), delta 3, accessory subunit  CKB - creatine kinase, brain  GSK3B - glycogen synthase kinase 3 beta  ASGR2 - asialoglycoprotein receptor 2  PRMT5 - protein arginine methyltransferase 5  TEAD2 - tea domain family member 2  NR2F6 - nuclear receptor subfamily 2, group f, member 6 |

^1^Enrichment p-value computed according to the mHG or HG model

^2^Correction of the p-value for multiple testing using the Benjamini and Hochberg method.

^3^Enrichment = (b/n) / (B/N); N: the total number of genes; B: the total number of genes associated with a specific GO term; n: the number of genes in the target set; b: the number of genes in the intersection.
