# Supplementary material for: Leishmania Infection Induces MicroRNA hsa-miR-346 in Human Cell Line-Derived Macrophages
Source: Front Microbiol. 2018 May 17;9:1019. doi: 10.3389/fmicb.2018.01019 (PMC5966562; doi:10.3389/fmicb.2018.01019)
Supplement: Supplementary file 3 [file Image_1.PDF]

Supplementary Figure S1

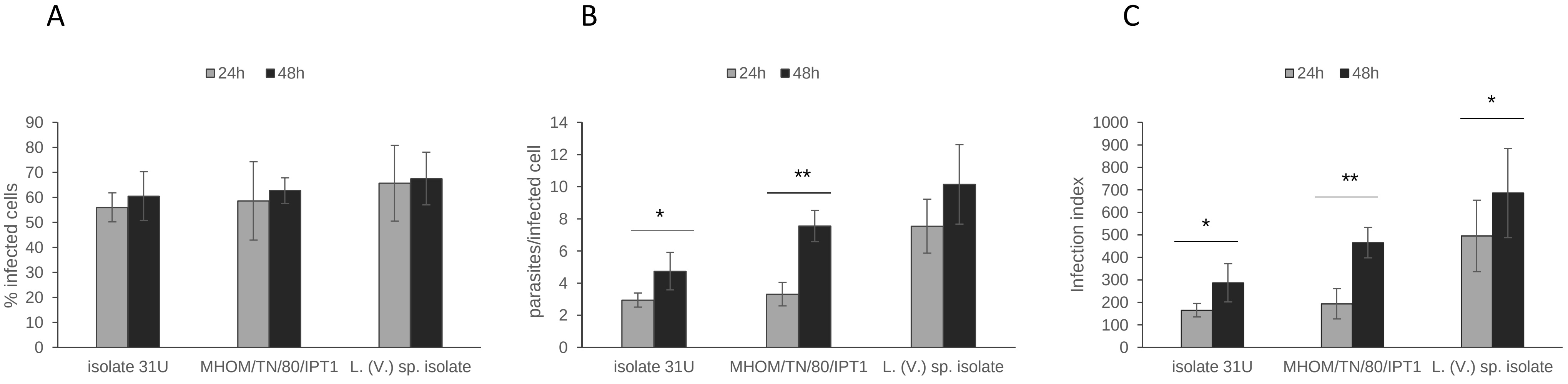

Supplementary Figure S1. Percentage of infected cells (A), number of parasites/infected cell (B) and infection index (C) in THP1-derived macrophages infected with L. (L.) infantum isolate 31U (n=5), MHOM/TN/80/IPT1 (n=7) and L. (V.) sp. isolate (n=8). *$p < 0.05$; **$p < 0.01$
