# Supplementary material for: Leishmania Infection Induces MicroRNA hsa-miR-346 in Human Cell Line-Derived Macrophages
Source: Front Microbiol. 2018 May 17;9:1019. doi: 10.3389/fmicb.2018.01019 (PMC5966562; doi:10.3389/fmicb.2018.01019)
Supplement: Supplementary file 4 [file Image_2.PDF]

Supplementary Figure S2

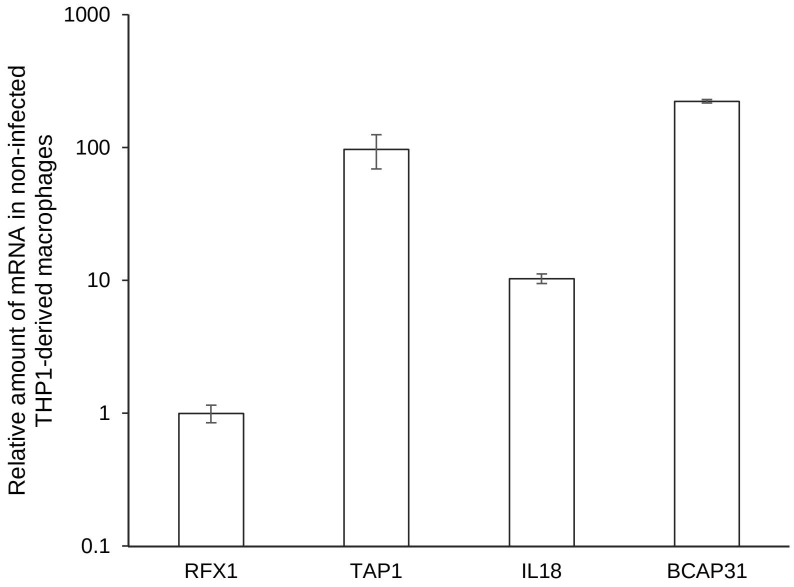

Supplementary Figure S2. The relative amounts of mRNA of RFX1 (n=4), TAP1 (n=10), IL18 (n=4), BCAP31 (n=4) was evaluated in non-infected THP-1-derived macrophages using ΔCt method.
