# Supplementary material for: Leishmania Infection Induces MicroRNA hsa-miR-346 in Human Cell Line-Derived Macrophages
Source: Front Microbiol. 2018 May 17;9:1019. doi: 10.3389/fmicb.2018.01019 (PMC5966562; doi:10.3389/fmicb.2018.01019)
Supplement: Supplementary file 5 [file Image_3.PDF]

Supplementary Figure S3

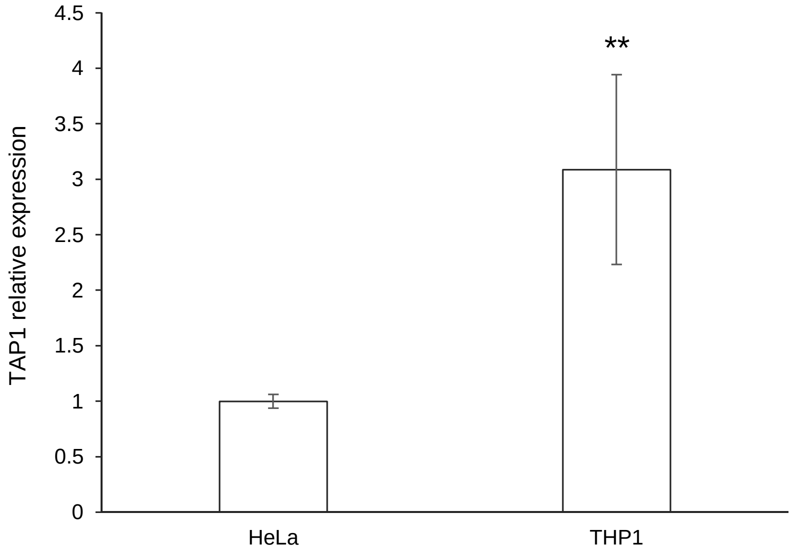

Supplementary Figure S3. TAP1 relative expression measured in HeLa cells (n=4) and THP1-derived macrophages (n=10) using ΔCt method. $^{**}p<0.01$
